# Children orphaned from COVID-19 in Thailand: maximize use of civil registration database for policies

**DOI:** 10.3389/fpubh.2023.1260069

**Published:** 2023-10-17

**Authors:** Viroj Tangcharoensathien, Sopon Iamsirithaworn, Jongjit Rittirong, Sanchai Techanimitvat, Patama Vapattanawong, Lucksana Apiratipanya, Thiphaphon Chanthama, Putthipanya Rueangsom

**Affiliations:** ^1^International Health Policy Program, Ministry of Public Health, Nonthaburi, Thailand; ^2^Department of Disease Control, Ministry of Public Health, Nonthaburi, Thailand; ^3^Institute for Population and Social Research, Mahidol University, Salaya, Nakhon Pathom, Thailand; ^4^Bureau of Registration Administration, Bangkok, Thailand

**Keywords:** orphan, COVID-19, pandemic, civil registration, Universal Health Coverage

## Abstract

Orphans, especially those who experience maternal loss at a young age, face significant long-term negative impacts on their lives and psychological well-being, extending beyond the age of 18. As of July 2023, the global death toll of COVID-19 has reached 6.9 million, leaving behind an unknown number of orphans who require immediate attention and support from policymakers. In Thailand, from April 2020 to July 2022, the total number of COVID-19-related deaths reached 42,194, resulting in 4,139 parental orphans. Among them, 452 (10.9%) were children under the age of five, who are particularly vulnerable and necessitate special policy attention and ongoing support. While the provision of 12 years of free education for all and Universal Health Coverage helps alleviate the education and health expenses borne by households supporting these orphans, the monthly government support of 2,000 Baht until the age of 18 is insufficient to cover their living costs and other education-related expenditures. We advocate for adequate financial and social support for COVID-19 orphans, emphasizing the importance of placing them with relatives rather than institutional homes. In the context of post-pandemic recovery, this perspective calls upon governments and global communities to estimate the number of orphans and implement policies to safeguard and support them in the aftermath of COVID-19.

## Introduction

In Thailand, as of July 2023, there have been 4.7 million COVID-19 infections and 34,371 related deaths. The death rate stands at 490 per million population, ranking 139th globally, indicating a relatively low rate (1). The pandemic has devastating impacts on population health and economic (2).

As of July 2023, the pandemic has claimed a minimum of 6.899 million lives worldwide, although this data may be subject to inaccuracies due to variations in mortality reporting by different countries. The relatively low COVID-19 infection and case fatality rates in Africa can be attributed to factors such as low testing rates, inadequate documentation of causes of death, a younger population, sufficient exposure to sunlight resulting in good vitamin D status, cross-immunity from other viruses including coronaviruses (3).

UNICEF defines an orphan as a child under 18 years of age who has lost one or both parents to any cause of death. Given the global mortality associated with COVID-19, it is crucial for society to comprehend the scale of orphanhood resulting from parents’ deaths linked to the virus. These orphans require immediate policy attention and comprehensive support.

### Long-term impacts of orphanhood

An orphan at a very young age, especially the loss of a mother, has a long-term impact on their lives and psychological well-being even beyond 18 years. Yearning for their mothers negatively affected their ability to develop coping strategies, which led to isolation, hopelessness, and fear of an uncertain future (4). Evidence confirms orphanhood increases the risk of stunting (5), delays child development, and exposes them to violence and exploitation (6–8).

The level of impact on orphans is determined by parental role and living context. In any case, losing a breadwinner in the household affects the livelihood of everyone, including the orphan (9). Government income support can mitigate the impacts (10), though nothing can substitute parenting function in support of early childhood development, which shapes biological and psychological structures and functions that affect health, well-being, and productivity throughout the life course of the orphans (11). Orphans living in institutions have an increased risk of negative health and developmental outcomes, demanding the need for deinstitutionalisation (12), and support to live with their own family relatives or fostered care homes (13).

### Orphans from COVID-19 pandemic

By May 1, 2022, a study estimates a minimum 10.5 million children affected by COVID-19-associated orphanhood globally. More orphan impacts in countries with lower vaccination rates and higher fertility rates. Children are at a higher risk of having lost a father than a mother and two out of every three affected children are between the ages of 11 and 18 (14). This study offers interactive visualization tool for global, regional, and country-level maps with orphan estimates (15).

A study (16) updated its previous estimates (17) in 21 countries regarding the number of COVID-19-associated orphan children. Over the 20 months between 1 March 2020 and 31 October 2021, there were 5 million COVID-19 deaths, resulting in 5.2 million children losing a parent or caregiver. Using an online COVID-19 orphanhood calculator, a study demonstrates that countries in the African region had the highest number of estimated parental orphans per death due to COVID-19 deaths among younger persons (18).

During the COVID-19 pandemic, social workers in Indonesia applied RapidPro (19) (a software application developed by UNICEF which captures data from the field) when they encountered children orphaned by COVID-19 for targeted support (20). Indonesia estimated that 25,430 children had lost one or both caregivers from COVID-19 between early 2020 and September 2021 (21). UNICEF in South Africa also estimated about 150,000 children were orphaned during the pandemic (22).

In the US, there were more than 250,000 children who have lost parents or caregivers to COVID-19. The ABC News reports the challenges, triumphs, and the way these orphans keep the memories alive (23, 24). In India, by 2021 it was estimated to have left behind more than 100,000 orphans from parental COVID-19 deaths (25).

By maximizing the use of the civil registration system, this Perspective estimates the size, age, and gender of orphans from the pandemic in Thailand. While we bring the issue of orphan from the pandemic to global policy agenda, we advocate Thai government for adequate support.

### Civil registration systems in Thailand

Thailand has continuously strengthened the civil registration system since the 1950s; it is now fully computerized for birth and death registration. By law, births must register with the local registration office within 15 days, while all deaths are within 24 h. Failure to comply with the law is subject to no more than a 1,000 Baht fine (US$ 33 at a 30 Baht exchange rate). The local registration offices record essential parameters nationwide online. All these are managed by the Bureau of Registration Administration (BORA).

Thailand’s civil registration system has matured, with high birth and death registration coverage. The National Statistical Office reported the completeness of birth and death registration increased from 96.7% of total births and 95.2% of total deaths in 2005–2006 to 99.4% and 99.3% in 2015–2016 (26). Despite high registration coverage, the cause of death is relatively poor, especially among those who died outside hospitals. Although the ill-defined codes reduced from 29.7% of deaths in 2013 to 24.3% in 2018, the proportion remains high (27). The interim strategy was applied by using verbal autopsy for non-hospital mortality (28, 29).

### Orphans from COVID-19 pandemic: Thailand direct estimate from civil registry

Using COVID-19 in-hospital mortality associated with COVID-19 registered with the civil registration office between April 2020 and July 2022, BORA estimated the total number of orphans by linking the citizen ID of all COVID-19 mortality with the nationwide household registration database and produced a number of orphans by age, gender, district, and province; and by parental, grand-parental and other relative deaths. Son or daughter younger than 18 living in the household with the deceased were tallied.

During this period, there were 42,194 in-hospital deaths associated with COVID-19, 53.8% male, 46.2% female, 26% below 60 years old, and 74% above 60 years old, see Table 1.

**Table 1:** Total in-hospital deaths associated with COVID-19, April 2020–July 2022, Thailand.

Age group	Male	Female	Total deaths	%
0–4	45	38	83	0.2%
5–9	19	13	32	0.1%
10–14	14	13	27	0.1%
15–19	31	31	62	0.1%
20–24	73	83	156	0.4%
25–29	183	121	304	0.7%
30–34	278	230	508	1.2%
35–39	441	337	778	1.8%
40–44	721	493	1,214	2.9%
45–49	1,029	702	1,731	4.1%
50–54	1,481	1,114	2,595	6.2%
55–59	1,977	1,500	3,477	8.2%
60–64	2,314	1,704	4,018	9.5%
65–69	2,699	2,097	4,796	11.4%
70–74	2,801	2,233	5,034	11.9%
75–79	2,574	2,177	4,751	11.3%
80+	6,011	6,617	12,628	29.9%
All age group	22,691	19,503	42,194	100.0%
Percent	53.8%	46.2%	100.0%	··

Bureau of Registration Administration.

**Figure 1:**
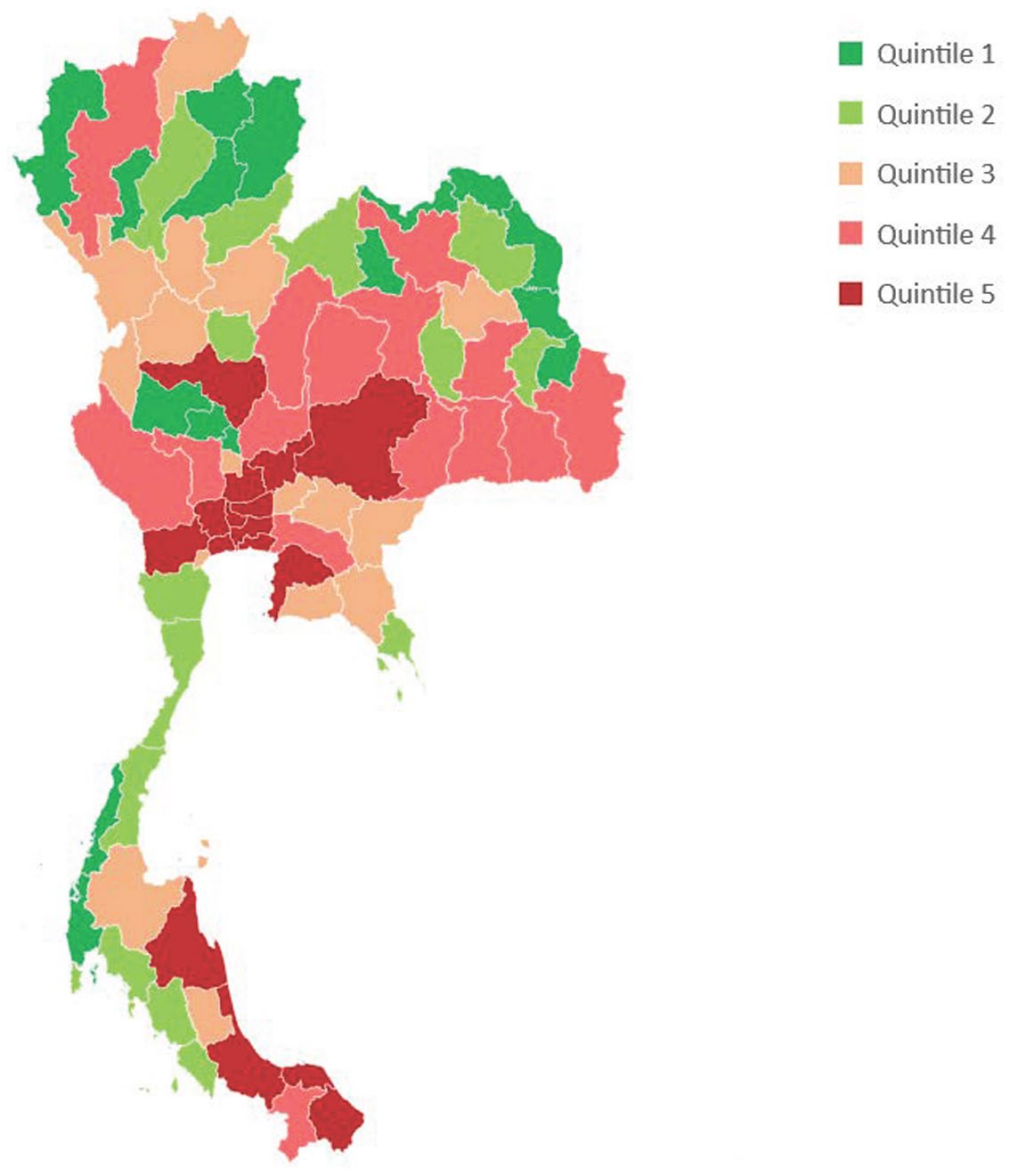
Total in-hospital deaths associated with COVID-19 by provincial quintiles.

Figure 1 describes COVID-19 in-hospital mortality associated with COVID-19 in each province classified by quintiles. Bangkok and a few provinces in central and southern regions are epi-centers of mortality.

From the 42,194 in-hospital mortality associated with COVID-19, we estimated 4,139 parental orphans, of which 2,094 (50.5%) were boys and 2,045 (49.5%) were girls. There were 452 under five years old orphans who lost their parents (48.2% boys, 51.8% girls) who are most vulnerable and require special policy support. Of the total 4,139 parental orphans, 2,719 (66%) were paternal, and 1,412 (34%) were maternal orphans, only 8 (0.2%) orphans lost both mothers and fathers.

We also estimated 6,507 orphans from the deaths of other relatives who lived in the same households and 47,422 grand-parental orphans. In total, there were 58,068 orphans attributable to the pandemic, see Table 2.

**Table 2:** Total orphans: parental, other relatives, and grand-parents, by gender and age groups.

	Parental orphan	Relative orphan	Grand-parental orphan	Total orphan	%
Male					
0–4 years	218	644	3,939	4,801	8.3%
5–9 years	438	903	6,233	7,574	13.0%
10–14 years	784	1,034	8,381	10,199	17.6%
15–18 years	654	712	5,682	7,048	12.1%
*Total male*	*2,094*	*3,293*	*24,235*	*29,622*	*51.0%*
Female					
0–4 years	234	627	3,856	4,717	8.1%
5–9 years	402	867	5,986	7,255	12.5%
10–14 years	795	1,054	7,950	9,799	16.9%
15–18 years	614	666	5,395	6,675	11.5%
*Total female*	*2,045*	*3,214*	*23,187*	*28,446*	*49.0%*
Both gender	4,139	6,507	47,422	58,068	100.0%
Percent	7.1%	11.2%	81.7%	100.0%	··

**Figure 2:**
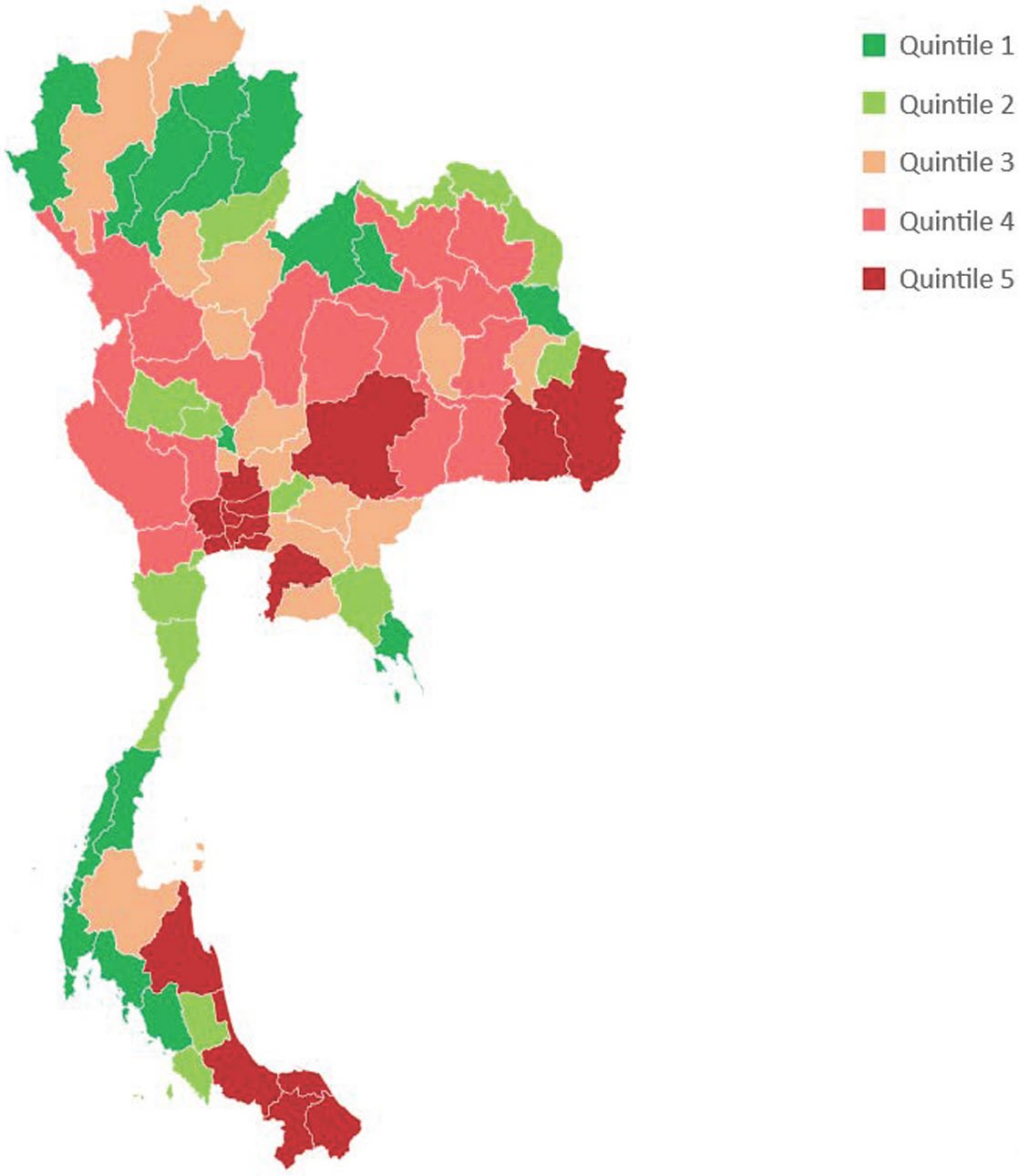
Parental orphans by province, sorted by quintile of the number of orphans in each province.

Figure 2 depicts parental orphans by provincial quintiles. Orphans concentrated in Bangkok and certain provinces in central, northeast, and southern regions were heavily affected by pandemic orphanages.

### Thailand orphans: government financial assistance policy

The Child Protection Act, B.E. 2,546 (30) defines an orphan as a child less than 18 years old whose father or mother has died. In response to the legal mandate provided by the Act, the Ministry of Social Development and Human Security provides monthly financial assistance to orphans from COVID-19, the amount of 2,000 Baht (US$ 67 at 30 Baht exchange rate) to the child’s own family or any person who provide care to them until the age of 18 years old. The Ministry’s policy advocates orphans to stay with their own families; however, if a foster family legally adopts them, there is no financial support to the foster family as all cost of providing welfare to the adopted child is the responsibility of the foster family. According to the policy, the least preferred choice is the institutional home managed by the Ministry.

We estimate the total budget required for all 4,139 parental orphans from the month of their orphanhood until they are 18. As we do not have data on which month of being orphan, we estimated based on two scenarios. First, pay for the second half of the first year of being an orphan (a 6-month payment). Second, pay for the whole first year (a 12-month payment). The total budget for Scenario 1 would be 617.1 million Baht (US$ 20.6 million) or an average of 170,053 Baht *per capita* orphan. Scenario 2 would cost 666.8 million Baht (US 22.2 million) or an average of 183,740 Baht *per capita* orphan. Geographical mapping by province facilitates the government in identifying orphans and providing support proactively. This budget size is minimal compared with the 21,411 million Baht Budget of the Ministry of Social Development and Human Security in the fiscal year 2022. Given Thailand’s upper-middle income country status, GNI *per capita* equals PPP$ 18,530 in 2021.

## Discussion

During the pandemic, the timely production of COVID mortality data from the civil registration system to support pandemic control policies has been challenging. To address this, the Ministry of Public Health Department of Disease Control (DDC) implemented additional weekly reports on COVID-19 mortality by requesting healthcare facilities to report online through the Provincial Health Offices. These reports were used to inform pandemic containment strategies and were publicly reported by the Centre for COVID-19 Situation Administration, chaired by the Prime Minister. However, the DDC mortality statistics suffer from incomplete reporting by both public and private hospitals, as they are focused on case management and maintaining essential health services. Therefore, we did not utilize the DDC database to estimate the number of orphans.

In this Perspective, we adopt the terminology used by the WHO, referring to COVID-19-associated mortality, as differentiating between dying from and dying with COVID-19 is challenging and requires histological diagnosis from autopsies (31, 32). However, all the in-hospital mortality cases included in our study were confirmed positive using RT-PCR.

As of August 2021, the provincial social development offices identified and provided financial support to 234 orphans through active searches (33). We recommend launching a comprehensive public campaign to increase awareness so that families with orphans can come forward and benefit from the available assistance. Concurrently, the provincial social development offices should continue their active search efforts.

Article 54 of the 2017 Constitution of Thailand mandates the government to provide 12 years of free education to all citizens, including three years for preschool age. However, this means that the government does not cover grades 10–12. While a comprehensive package of health services has been provided free of charge through the Universal Health Coverage system in Thailand since 2002, resulting in a low prevalence of catastrophic health spending (34), the financial burden on households for food, transportation, living costs, and other essentials remains substantial. The current monthly support of 2,000 Baht is inadequate and fails to account for future inflation. Additionally, full payment for education in grades 10–12 is required to receive government support. We strongly advocate for a significant revision of the payment rate in line with these observations.

This Perspective brings the issue of orphans to the forefront of the global policy agenda and highlights the potential of utilizing civil registration databases, specifically household registries, for an accurate count of orphans resulting from COVID-19. This approach incurs no additional costs compared to surveys and provides more precise data than modelling. The publication of the number of orphans by province, along with public awareness campaigns through social media, can garner media and community attention, encouraging support for orphans.

However, it is important to note that this approach is only feasible in countries where birth and death registration systems are complete and fully computerized. Unfortunately, many Asian and African countries lack the capacity for adequate civil registration systems. We recommend strengthening civil registration systems in low- and middle-income countries, in line with the commitment to SDG16 and indicator 16.9, which aims to provide legal identity, including birth registration, for all by 2030. Efforts should be made to identify orphans and provide timely comprehensive support.

Civil registration records births, deaths, marriages, divorces, and legal residency of citizens (35), all of which ensure citizen rights and access to education and health services, and effective legal protection (36). It is the best data source to monitor progress towards numerous SDG targets and indicators (37). The by-product of civil registration is the direct estimate of orphans.

In the context of poorly developed mortality registration system in low- and middle-income countries where direct estimate of orphans is not feasible, we propose a two-pronged policy strategy. While governments strengthen civil registration in long term, they can introduce social protection measures such as income and education support to orphans. Government can create public awareness to these policies in the communities, and civil society can actively identify families with orphans to avail of these supports.

The social support, either in cash or in kind, should be adequate and determined by government’s fiscal space. Citizen’s engagement in the policy formulation not only increases the effectiveness and uptake of these social protection measures (38), they also inform implementation practicability (39). Further, government can mobilize social capital embedded in the community through their social network and cohesion (40) to provide additional psychosocial support to the orphans (41).

The World Bank reported uneven economic recovery across countries; low-income countries had the least GNI growth partly due to the slow pace of vaccination (42). By 2021, the debt-service payment by countries eligible in the International Development Association on long-term public and publicly guaranteed external debt totaled $46.2 billion—equivalent to 10.3% of their exports of goods and services (increased from 3.2% in 2010) and 1.8% of their gross national income (up from 0.7% in 2010) (43). In the fiscal crunch and competing priorities between different sectors, governments, especially in countries with large size of orphan living in vulnerable situation, need to prioritize health, education, and wellbeing for orphans through multi-sectoral actions and community engagement in order to mitigate the long-lasting impacts.

Our figures may have certain limitations of potential under-estimate, such as under-reporting of the COVID-19 related deaths due to stigmatization, undiagnosed, and inability to access treatment.

## Data availability statement

The data analyzed in this study is subject to the following licenses/restrictions: the dataset is available upon request. Requests to access these datasets should be directed to Department of Disease Control, Ministry of Public Health, Thailand and Bureau of Registration Administration, Thailand.

## Author contributions

VT: Conceptualization, Investigation, Methodology, Project administration, Resources, Supervision, Writing – original draft, Writing – review & editing. SI: Validation, Writing – review & editing. JR: Conceptualization, Writing – review & editing, Methodology. ST: Data curation, Writing – review & editing. PV: Conceptualization, Writing – review & editing, Methodology. LA: Data curation, Writing – review & editing. TC: Visualization, Writing – review & editing, Formal analysis. PR: Formal analysis, Writing – review & editing, Visualization.
